# Fibro-osseous pseudotumor of the digit presenting as an ulcerated lesion: a case report

**DOI:** 10.1186/1755-7682-7-4

**Published:** 2014-01-09

**Authors:** Atif Ali Hashmi, Naveen Faridi, Muhammad Muzzammil Edhi, Asad Jafri, Mehmood Khan

**Affiliations:** 1 Department of Histopathology, Liaquat National Hospital and Medical College, Karachi, Pakistan; 2 Liaquat National Hospital and Medical College, Karachi, Pakistan; 3 Dhaka Medical College, Dhaka, Bangladesh

**Keywords:** Fibro osseous pseudotumor, Myositis ossificans

## Abstract

Fibro-osseous pseudotumor of the digit is rare benign lesion of subcutaneous tissue which is thought to be reactive process as a result of repeated trauma. We report a case of an ulcerated lesion of skin of middle finger, clinically thought to be leishmaniasis which after punch biopsy followed by excision turned out to be fibrosseous pseudotumor. Diagnosis of fibro-osseous pseudotumor requires immense precision as it can clinically mimic unungal exostosis and can sometimes be misinterpreted clinically and radiologically as myositis ossificans. We suggested an algorithimic approach for histopathologic assessment of fibro-osseous soft tissue lesions with evaluation of both stromal and osseous components. Bland fibroblastic stroma, mature osseous component with prominent osteoblastic rimming and absence of zonation pattern will support the diagnosis of fibro-osseous pseudotumor especially if located at a superficial and distal location.

## Background

Ossifying lesions of soft tissue are fairly uncommon. However they may be a cause of concern to the clinicians as aggressive bone forming tumors like extraskeletal osteosarcoma is one of the differential diagnosis [1]. Fibro-osseous pseudotumor of the digit is also noteworthy due to its odd location, i.e. tips of fingers and toes. To date only a few cases have been reported in the literature. There is no endemic area or familial clustering. In addition there is no racial or ethinic predilection. To our knowledge this is the first case reported from Pakistan. There is slight female predilection and is thought to be a reactive process [2-4].

In our opinion, early diagnosis and treatment (resection) of fibro-osseous pseudotumor is important due to three reasons. Firstly, as this is a rare entity, potential for misdiagnosis and overtreatment is high. As in our case it was misdiagnosed as an infectious process, while in other cases it was thought of a vascular lesion. Secondly although no case till date of malignant transformation of fibro-osseous pseudotumor is described, but a definite link between long standing chronic inflammatory or irritative lesions and neoplasia exists, therefore early excision is necessary. Lastly as this lesion is located superficially in dermis, therefore chances of ulceration and secondary infections are high necessitating early treatment.

Leishmaniasis is endemic in some rural parts of Pakistan. It usually presents as a papule which later converts to nodule and can ulcerate. On the other hand fibro-osseous pseudotumor presents as a hard subcutaneous nodule, which can also ulcerate mimicking leishmaniasis especially in endemic areas. A simple cytologic test looking for LD bodies can help in differentiation. In difficult cases a punch biopsy can lead to a definite diagnosis.

## Case presentation

Our case is that of a 32 year old male, who had a small dermal nodule on the dorsal aspect of middle finger for three years. There was no associated pain, joint swelling, redness or restriction of joint mobility. The patient did not recall any specific history of trauma. Later on the lesion becomes ulcerated due to which he seek medical attention. The patient was laborer by occupation and belonged to the rural part of the country. The initial clinical suspicion was leishmaniasis, due to which a punch biopsy was done. The biopsy revealed a dermal fibro-osseous lesion and therefore excision was advised. The lesion measured 1.7 × 1.5 × 1.2 cm and no connection with underlying bone was demonstrated. Post-operatively, the wound healed quickly. The patient was followed for one year, however no recurrence was noted.

### Pathology

**Figure 1:**
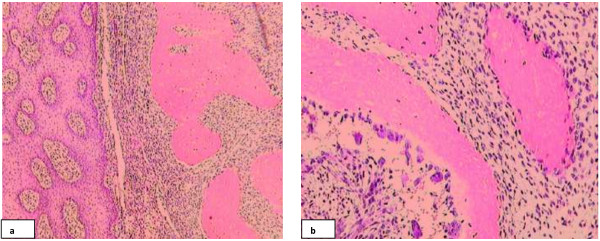
**H and E sections fibro-osseous lesion in dermis. a)** lesion composed of fibrovascular stroma and mature bony trabeculae. **b)** high power revealed prominent osteoblastic rimming and bland stromal cells.

Grossly the specimen consisted of a skin covered fragment with a firm nodular lesion in dermis measuring 1.5 cm in maximum dimention. Microscopic sections examined revealed biphasic fibro-osseous lesion present in the dermis, composed of stromal and osseous components. Overlying skin appeared midly hyperplastic and borders of the lesion were well circumscribed. Stromal component of the tumor is composed of bland spindle shaped cells with interspersed blood vessels. Stromal cells appear fibroblastic with elongated nuclei, eiosinophillic cytoplasm and indistinct cell borders. They show minimal nuclear atypia and pleomorphism. Osseous component was formed by mature bony trabeculae with prominent osteoblastic rimming. Zonation pattern was not evident in the lesion. No mitotic activity was noted. The lesion appeared completely excised (Figure 1).

**Figure 2:**
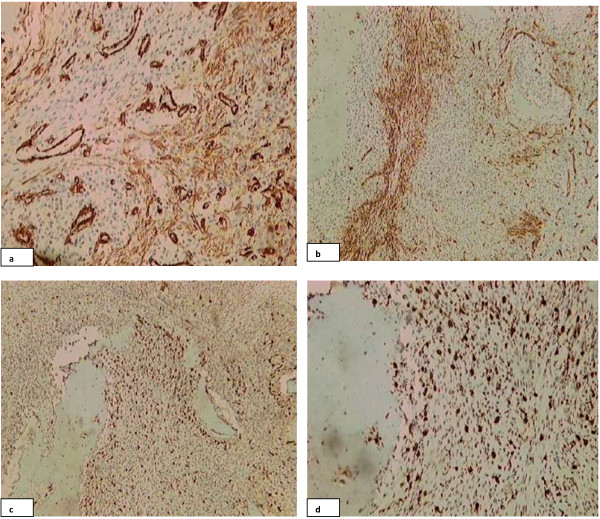
**Photomicrography after applying immunohistochemical stains revealing. a)** ASMA stain highlighting blood vessels with focal positivity in stromal cells. **b)** CD34 stain showing focal positivity in stromal cells. **c)** and **d)** S100 stain also revealed focal staining in stromal cells.

Immunohistochemical stains were performed on the section by DAKO envision method using antibodies against pancytokeratin (CKAE1/AE3), ASMA, S100 and CD 34. Stromal cells were negative with cytokeratin, however focal positivity was noted with ASMA, CD 34 and S100 stains (Figure 2).

## Discussion

Fibro-osseous pseudotumor of the digit is a rare benign ossifying lesion which had been called in the past by various names like florid reactive periostitis, parosteal fasciitis and fasciitis ossificans [5-7]. To our knowledge, no case of fibro-osseous pseudotumor was reported from our country to date.

Pathogenesis of fibro-osseous pseudotumor is thought to be related to repeated trauma to the area, however a specific history of antecedent trauma was revealed in a small number of cases [3]. In our case, although there was no preceding trauma history, but as the patient was a laborer, therefore repeated trauma was un-avoidable.

Main pathologic differentials to this entity are myositis ossificans, subungal exostosis and extraskeletal osteosarcoma. Fibro-osseous pseudotumor is closely related to myositis ossifians and some authors regard it as a cutaneous counterpart of myositis ossificans [8]. However, myositis ossificans usually occur after trauma, in the deeper aspect of proximal soft tissues, and histopathologically show a typical zonation pattern [9]. Subungal exostosis may clinically simulate fibros-osseous pseudotumor, but radiologic connection to the underlying bone and histopathologic demonstration of fibrocollageous cap separate out the two entities [10]. Finally extraskeletal osteosarcoma should always be ruled out, however it shows destructive stromal invasion, obvious cytologic atypia and immature ostoid directly formed by tumor cells [11].

We found focal positivity of ASMA, S100 and CD 34 immunostains in our case. Other authors found positivity with ASMA, however staining with S100 and CD34 was not demonstrated [8].

Prognosis of fibro-osseous pseudotumor is good with complete excision being curative without evidence of local recurrence [3,9], as was seen in our case. Although histopathologic differentials are limited, but we suggest an algorithimic approach to diagnose fibro-osseous soft tissue lesions. First the stromal component should be evaluated for evidence of malignancy including nuclear atypia, pleomorphism, mitotic activity and growth pattern. If the stromal component of the tumor appear malignant, then the differentials include osteosarcoma and other dedifferentiated sarcomas with heterologous differentiation. On the other hand, if the stroma appear fibroblastic type then osseous component should be further uncovered for maturation and osteoblastic rimming. With mature osteoid and prominent osteoblastic rimming, the main differential would be fibro-osseous pseudotumor and myositis ossificans. Distal and superficial location with absence of zonation pattern will favor the diagnosis of fibro-osseous pseudotumor while deep lesions in proximal location and zonation pattern will suggest the diagnosis of myositis ossificans.

## Conclusion

Fibro-osseous pseudotumor is a rare benign reactive condition diagnosis of which requires immense precision as it can clinically mimic unungal exostosis and can sometimes be misinterpreted clinically and radiologically as myositis ossificans. We suggested an algorithimic approach for the histopathologic interpretation of soft tissue fibro-osseous lesions.

## Consent

Patient has given their informed consent for the case report to be published.

## Competing interest

The authors declare that they have no competing interests.

## Authors’ contributions

AAH: main author of manuscript, have made substantial contributions to conception, design and acquisition of data. NF: have given final approval of the version to be published. MME: involved in drafting the manuscript and revising it critically for important intellectual content. AJ: have given final approval of the version to be published. MK: involved in drafting the manuscript and revising it critically for important intellectual content. All authors read and approved the final manuscript.

## References

[B1] Nishio J Iwasaki H Soejima O Naito M Kikuchi M Rapidly growing fibro-osseous pseudotumor of the digits mimicking extraskeletal osteosarcoma J Orthop Sci 2002 7 3 410 413 10.1007/s007760200070 12077672

[B2] Dupree WB Enzinger FM Fibro-osseous pseudotumor of the digits Cancer 1986 58 2103 2109 10.1002/1097-0142(19861101)58:9<2103::AID-CNCR2820580923>3.0.CO;2-C 3463398

[B3] Moosavi CA Al-Nahar LA Murphey MD Fanburg-Smith JC Fibroosseous pseudotumor of the digit: a clinicopathologic study of 43 new cases Ann Diag Pathol 2008 12 21 28 10.1016/j.anndiagpath.2007.02.001 18164411

[B4] Tan KB Tan SH Aw DC Lee YS Fibro-osseous pseudotumor of the digit: presentation as an enlarging erythematous cutaneous nodule Dermatol Online J 2010 16 7 21199633

[B5] Spjut HJ Dorfman HD Florid reactive periostitis of the tubular bones of the hands and feet. A benign lesion which may simulate osteosarcoma Am J Surg Pathol 1981 5 5 423 433 10.1097/00000478-198107000-00002 6945056

[B6] McCarthy EF Ireland DC Sprague BL Bonfiglio M Parosteal (nodular) fasciitis of the hand. A case report J Bone Joint Surg Am 1976 58 5 714 716 1064594

[B7] Kwittken J Branche M Fasciitis ossificans Am J Clin Pathol 1969 51 2 251 255 4974865 10.1093/ajcp/51.2.251

[B8] Sleater J Mullins D Chun K Hendricks J Fibro-osseous pseudotumor of the digit: a comparison to myositis ossificans by light microscopy and immunohistochemical methods J Cutan Pathol 1996 23 4 373 377 10.1111/j.1600-0560.1996.tb01313.x 8864927

[B9] De Silva MV Reid R Myositis ossificans and fibroosseous pseudotumor of digits: a clinicopathological review of 64 cases with emphasis on diagnostic pitfalls Int J Surg Pathol 2003 11 3 187 195 10.1177/106689690301100305 12894350

[B10] Dave S Carounanidy U Thappa DM Jayanthi S Subungual exostosis of the thumb Dermatol Online J 2004 10 1 15 15347497

[B11] Tan KT Idowu OK Chandrasekar CR Yin Q Helliwell TR Extraskeletal osteosarcoma of the hand Hand (N Y) 2012 7 1 124 126 10.1007/s11552-011-9371-3 23450980 PMC3280379

